# Microbiological point of care testing before antibiotic prescribing in primary care: considerable variations between practices

**DOI:** 10.1186/s12875-016-0576-y

**Published:** 2017-01-26

**Authors:** Steffen Haldrup, Reimar W. Thomsen, Flemming Bro, Robert Skov, Lars Bjerrum, Mette Søgaard

**Affiliations:** 1 Department of Clinical Epidemiology, Aarhus University, Olof Palmes Alle 43-45, 8200 Aarhus N, Denmark; 2 Department of General Practice, Institute of Public Health, Aarhus University, Aarhus, Denmark; 3 Antimicrobial Resistance Reference Laboratory and Surveillance Unit, Department of Microbiology and Infection Control, Statens Serum Institut, Copenhagen, Denmark; 4 Section and Research Unit of General Practice, Department of Public Health, Copenhagen University, Copenhagen, Denmark

**Keywords:** Point-of-care, Antibiotic, Infection, General practice

## Abstract

**Background:**

Point-of-care testing (POCT) in primary care may improve rational antibiotic prescribing. We examined use of POCT in Denmark, including patient- and general practitioner (GP)-related predictors.

**Methods:**

We linked nationwide health care databases to assess POCT use (C-reactive protein (CRP), group A streptococcal (GAS) antigen swabs, bacteriological cultures, and urine test strips) per 1,000 overall GP consultations, 2004–2013. We computed odds ratios (OR) of POCT in patients prescribed antibiotics according to patient and GP age and sex, GP practice type, location, and workload.

**Results:**

The overall use of POCT in Denmark increased by 45.8% during 2004–2013, from 147.2 per 1,000 overall consultations to 214.8. CRP tests increased by 132%, bacteriological cultures by 101.7% while GAS swabs decreased by 8.6%. POCT preceded 28% of antibiotic prescriptions in 2004 increasing to 44% in 2013. The use of POCT varied more than 5-fold among individual practices, from 54.9 to 394.7 per 1,000 consultations in 2013. POCT use varied substantially with patient age, and males were less likely to receive POCT than females (adjusted OR = 0.75, 95% CI 0.74-0.75) driven by usage of urine test strips among females (18% vs. 7%). Odds of POCT were higher among female GPs and decreased with higher GP age, with lowest usage among male GPs >60 years. GP urban/rural location and workload had little impact.

**Conclusion:**

GPs use POCT increasingly with the highest use among young female GPs. In 2013, 44% of all antibiotic prescriptions were preceded by POCT but testing rates vary greatly across individual GPs.

## Background

As much as 50% of antibiotic use in human medicine may be either unnecessary or inappropriate across all health care settings [1–3]. For instance, acute bronchitis accounts for approximately 80% of lower respiratory tract infections and despite guidelines, randomized controlled trials, and meta-analyses showing little or no benefit from antibiotics [4, 5], up to 80% of patients consulting for this condition are prescribed antibiotics [6–9].

In Denmark, general practitioners (GPs) account for more than 80% of the total antibiotic use [10]. To reduce ineffective and unnecessary antibiotics the Danish National Board of Health in 2012 issued a guideline that encourages rational antibiotic prescribing (i.e., use of narrow spectrum antibiotics), and to only prescribe antibiotics when necessary based on clinical and microbiological examination [11]. Diagnostic uncertainty increases the risk of unnecessary antibiotic prescribing [6, 12], and a key to rational prescribing is to perform point-of-care testing (POCT) [13]. POCT is defined as medical diagnostic testing at or near the site of care [14], and use of POCT such as enzyme immunoassay kits (e.g., group A streptococcal (GAS) antigen), measurement of C-reactive protein (CRP), urine test strips and bacteriological cultures may reduce diagnostic uncertainty [15–17] and thereby antibiotic prescribing [17–23]. Thus, POCT may contribute to safely withholding antibiotics from patients who most probably would not benefit from antibiotic treatment. Nonetheless, while there is a large body of literature on prescribing patterns among GPs, few studies have examined the use of POCT in relation to antibiotic prescribing [16, 23, 24]. Studies from Sweden and Switzerland have shown that approximately 42% of patients consulting a GP for an acute respiratory tract infection receive a CRP-test [16, 25]. Older patients, those with higher education, and those with more discomfort are more likely to receive testing [16]. Another study showed that physicians were less likely to perform streptococcal tests in children with pharyngitis at the end of the week (Thursday and Friday) (adjusted relative risk 0.75, 95% confidence interval (CI) 0.66-0.87) compared with the first days of the week [26]. In line with this, a US study showed that streptococcal testing rates varied from 59% to 83% among different health plans for children with pharyngitis who were prescribed antibiotics [27]. Still, there is limited population-based information on the prevalence and time trends of use of POCT before antibiotic prescribing in primary care and about what characterizes patients and GPs who use POCT. Information on predictors of POCT may help to identify interventions to improve the efficient use of antibiotics. We therefore undertook a nationwide population-based study to examine the use of POCT in relation to antibiotic prescribing in the Danish primary health care sector in 2004–2013, and investigated patient and GP-related predictors for use of POCT.

## Methods

### Setting and study population

This population-based cross-sectional study was based on the entire Danish population with approximately 5,5 million residents between 2004 and 2013. The Danish healthcare system provides tax-supported health care to all residents, guaranteeing free access to hospitals, primary medical care, and partial reimbursement for prescribed medications, including most antibiotics. A unique central personal registration number, assigned to all Danish residents at birth or upon immigration, is used to record health care services in various nationwide registries, allowing unambiguous linkage among registries [28]. The current nationwide study is based on information from the Danish National Health Insurance Service Registry (DNHSR) [29], the Danish National Health Service Prescription Database (DHSPD) [30], and data provided by Danish Regions (www.regioner.dk).

### POCT and antibiotic prescriptions

We obtained information on POCT and number of GP consultations through the DNHSR. Except for 2% of the population, all Danes are enlisted with a particular general practice of their choice and all services provided to this population are recorded through activity codes in the DNHSR which contains data collected from health contractors in primary care since 1990 [29]. The DNHSR include information about patients (e.g., civil registration (CRS) number, date of entering present general practice, present and previous general practice number), the general practices (including a unique practice identification number which we used to ascertain that only GPs were included, and practice type (single-handed vs. partnership)), and health services (type of consultation (ordinary, telephone, home visit, or email) and any laboratory tests performed (including CRP measurement, GAS antigen throat swabs, bacteriological cultures, urine test strips and microscopy). Each laboratory test has a unique four-digit code and is a health service for which the GP is compensated. We utilized these four digit codes, along with the six digit practice identification number, to identify all POCT performed by GPs between 2004 and 2013.

We then obtained information on all filled antibiotic prescriptions in Denmark during 2004–2013 through the DHSPD [30]. This database records the patients’ personal identifier, date of dispensing, and the type and quantity of drug prescribed (according to the Anatomical Therapeutic Chemical (ATC) Classification System) each time a prescription is redeemed at any Danish pharmacy. For all antibiotic prescriptions, we ascertained whether the patients had POCT performed within the preceding 14 days of filling the antibiotic prescription.

### Patient characteristics

We categorized patients according to sex, age (0–4, 5–9, 10–14, 15–19, 20–39, 40–64, 65–79, and ≥80 years), and prescribed antibiotics categorized as; tetracycline (J01AA), beta-lactamase sensitive penicillin (J01CE), penicillin with extended spectrum (J01CA), beta-lactamase resistant penicillin (J01CF), combinations of penicillin incl. beta-lactamase inhibitors (J01CR), sulphonamide and trimethoprim (J01E), macrolide, lincosamide and streptogamin (J01F), quinolone (J01MA), and other antibiotics (J01D, G, X).

### GP characteristics

Through the DNHSR, we obtained data on the GP’s annual number of ordinary consultations per 1,000 registered patients as proxy for GP workload (categorized in quintiles as <5675, 5675–6354, 6355–7189, >7190) [31], type of practice (single-handed versus partnership practice), and geographical location of practice (health care administrative region, city size (<5000 inhabitants or ≥5000 inhabitants). For single-handed practices we further obtained information about the GP’s age (<41, 41–50, 51–60, and ≥60 years) and sex from Danish Regions. This information was not available for partnership practices because the six-digit practice identifier is applied to the practice and not the individual GP. We restricted the study to GPs with at least 500 registered patients to reduce the impact by inactive GPs on the observed variation.

### Statistical analysis

We computed the annual prevalence of use of any POCT and of specific tests per 1,000 overall consultations in general practice, and estimated the prevalence proportion of antibiotic prescriptions preceded by POCT. We truncated the data at the 1st and 99th percentiles due to ambiguous rates (outliers). To compare the rates in different years and across different GPs, we standardized the rates to the age- and sex distribution of the population of Denmark as of January 1 2013 (obtained from statistics Denmark), using direct standardization. Subsequently, we used logistic regression to compute crude and adjusted odds ratios (OR) with 95% CIs for use of POCT prior to antibiotic prescribing according to patient age and sex, practice location (geographical region and city size) as well as the GP’s age, sex and workload. The predictors were mutually adjusted. As the prevalence of pre-antibiotic POCT changed over time, we restricted analyses of predictors to 2013 in order to describe predictors of POCT in the most recent year. Analyses of the impact of GP age and sex were restricted to single-handed practices as this information was not available for partnership-practices. Data management and statistical analysis was conducted in SAS version 9.2/9.4 (SAS Institute, Cary, NC) and STATA 14 (Stata Corp., College Station, TX, USA).

## Results

From 2004 to 2013, 27,267,874 antibiotic prescriptions were registered in the DHSPD. Of these, we excluded 5,496,924 prescriptions issued by other authorities than GPs in the primary health care, for instance doctors from the emergency service and specialists, and 1,608,012 prescriptions due to incomplete data, e.g., missing information about the issuing GP. Thus, the study included 20,162,938 antibiotic prescriptions issued by 2,021 practices to 4,434,916 patients. A total of 1,051 (52%) of the GPs worked in single-handed practices, and 970 (48%) constituted partnership practices. The usage of POCT before antibiotic prescribing amounted to 7,344,586 tests.

### Use of POCT according to type of prescribed antibiotics

**Fig. 1:**
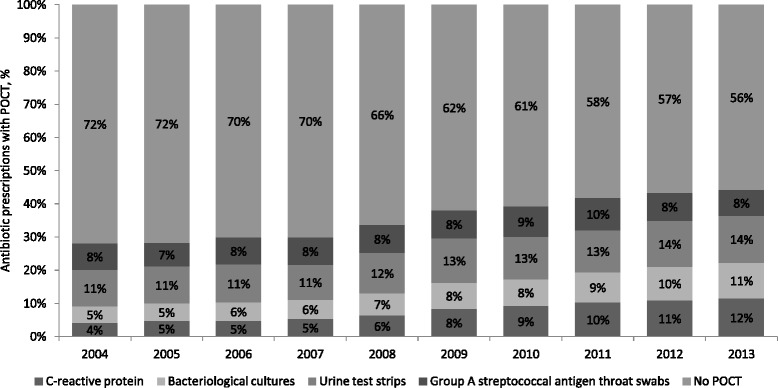
Proportion of antibiotic prescriptions with and without a preceding microbiological point-of-care test (POCT) according to type of test

**Fig. 2:**
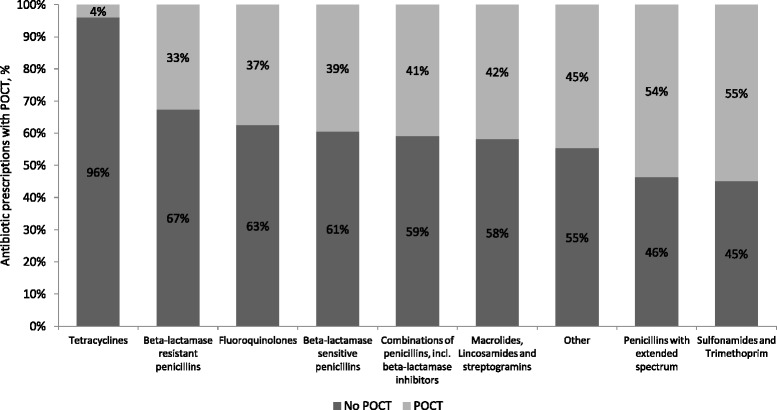
Proportion of antibiotic prescriptions with and without a preceding microbiological point-of-care test (POCT) in 2013 according to type of prescribed antibiotic

In 2004, 28% of the antibiotic prescriptions were preceded by POCT compared with 44% in 2013, corresponding to a 45% increase in the use of POCT prior to antibiotic prescribing (Fig. 1). The use of POCT differed substantially by type of prescribed antibiotic. For example, in 2013 the proportion of antibiotic prescriptions preceded by POCT varied from 4% for tetracycline to 44% for sulphonamide and trimethoprim prescriptions (Fig. 2).

### Age and sex standardised rates of POCT per 1,000 overall consultations

**Fig. 3:**
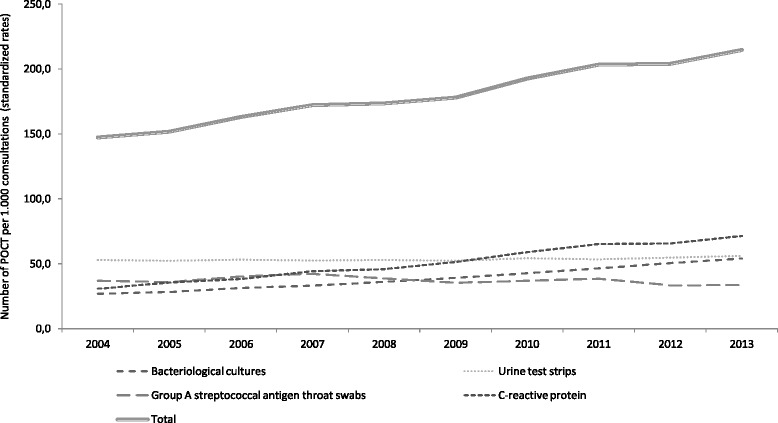
Age- and sex standardized rates of microbiological point-of-care testing in individuals prescribed antibiotics and overall volume of prescribed antibiotics in general practice, 2004–2013

The overall age and sex standardised prevalence rate of POCT was 147.2 per 1,000 consultations in 2004 and increased by 45.8% reaching 214.8 tests per 1,000 consultations in 2013. However, the use of individual POC tests differed markedly over time (Fig. 3). With 53.0 tests per 1,000 consultations urine test strips was by far the most frequently used test in 2004 and the usage increased by 5.5% ending at 55.9 per 1,000 consultations in 2013. Concurrently, CRP measurements increased by 132.0% (from 30.7 per 1,000 to 71.3 per 1,000) and bacteriological cultures by 101.7% (from 26.8 per 1,000 to 54.0 per 1,000). In 2013, CRP was the most frequently used test before antibiotic prescribing. In comparison, the use of GAS antigen swabs decreased by 8.6%, from 36.8 per 1,000 consultations in 2004 to 33.6 in 2013.

Rates of POCT use among the 2045 GPs varied by approximately a 5-fold, from 54.9 to 394.7 per 1,000 consultations in 2013 (median = 203, interquartile range (IQR) = 167–241) and even more for the individual tests; bacteriological cultures varied by more than 30-fold from 4.1 to 123.8 per 1,000 consultations (median = 48, IQR =32-67); urine test strips by almost a 12-fold from 11.6 to 121.3 per 1,000 (median =51, IQR =43-64); GAS antigen tests by more than 38-fold from 2.5 to 94.8 (median = 31, IQR = 24–42) and CRP measurement from 0.00 to 183.2 per 1,000 consultations (median = 63, IQR = 42–89).

### Predictors of POCT use according to patient and GP characteristics

Table 1 shows patient- and GP-related predictors of POCT prior to antibiotic prescribing in 2013. The proportion of patients with POCT varied from 33% in children aged 0–4 years to 59% in teenagers aged 15–19 years, equivalent to an adjusted OR of 2.14 (95% CI 2.10-2.17). The usage of the specific tests varied greatly by age, for example 31% of children aged 5–9 years were tested for GAS antigen prior to antibiotic prescribing compared with only 1% of patients aged 65–79 years, whereas the use of urine test strips increased with age. Use of bacteriological cultures and CRP measurement also varied with age but less pronounced. Compared with women, men were less likely to receive a POCT (adjusted OR = 0.75, 95% CI 0.74-0.75). This difference was primarily driven by higher usage of urine test strips among females (18% in females vs. 7% in males), while the use of other tests differed little by sex.

**Table 1:** Patient- and GP-related predictors of use of point-of-care (POC) testing prior to antibiotic prescribing in Denmark, 2013

Characteristic	Number of antibiotic prescriptions	Proportions of POC testing					OR for POC testing	
		POC test overall	Bacteriological cultures	Urinary test trips	Streptococcal antigen throat test	C-reactive protein	Crude OR (95% CI)	Adjusted^a^ OR (95% CI)
Patient-related predictors								
Patient age, years								
0–4	129,184	33%	5%	2%	19%	7%	1.00 (reference)	1.00 (reference)
5–9	78,301	50%	8%	6%	31%	4%	1.99 (1.96–2.03)	1.98–(1.94–2.02)
10–14	45,288	47%	10%	6%	26%	6%	1.81 (1.77–1.85)	1.80 (1.77–1.84)
15–19	91,909	52%	15%	15%	15%	7%	2.23 (2.19–2.26)	2.14(2.10–2.17)
20–39	411,473	49%	14%	12%	12%	10%	1.95 (1.92-1.97)	1.85 (1.83–1.88)
40–64	620,369	42%	9%	13%	5%	15%	1.44 (1.42-1.46)	1.41(1.39-1.43)
65–79	420,459	46%	11%	19%	1%	15%	1.75 (1.72-1.77)	1.73 (1.70-1.75)
≥ 80 years	272,590	41%	11%	22%	0%	8%	1.38 (1.36-1.40)	1.33 (1.31-1.35)
Patient sex								
Female	1,320,004	47%	11%	18%	7%	11%	1.00 (reference)	1.00 (reference)
Male	749,569	39%	10%	7%	9%	13%	0.73 (0.73-0.73)	0.75 (0.74-0.75)
Geographical region of practice location								
Capital Region	597,406	45%	12%	12%	10%	10%	1.00 (reference)	1.00 (reference)
Central Denmark	428,874	40%	11%	13%	6%	11%	0.84 (0.83-0.85)	0.81 (0.80-0.82)
North Denmark Region	222,702	49%	11%	16%	7%	15%	1.19 (1.18-1.20)	1.19 (1.18-1.20)
Region Zealand	318,353	43%	8%	17%	7%	10%	0.93 (0.92-0.93)	0.91 (0.91-0.92)
Region of Southern Denmark	502,238	46%	10%	15%	8%	13%	1.07 (1.06-1.07)	1.04 (1.03-1.05)
City size								
< 5,000 inhabitants	1,820,020	44%	11%	14%	8%	11%	1.00 (reference)	1.00 (reference)
≥ 5,000 inhabitants	249,553	44%	9%	15%	7%	13%	1.02 (1.01-1.03)	1.01 (1.01-1.01)
GP-related predictors								
GP age and sex (for single-handed practices)^b^								
< 41, female	11,833	47%	12%	14%	10%	12%	1.00 (reference)	1.00 (reference)
41-50, female	51,698	49%	13%	15%	10%	12%	1.07 (1.01-1.10)	1.06 (1.02-1.11)
51-60, female	70,474	47%	13%	14%	9%	11%	0.99 (0.97-1.05)	1.01 (0.95-1.03)
> 60, female	56,098	43%	11%	12%	10%	10%	0.83 (0.81-0.88)	0.84 (0.80-0.86)
< 41, male	14,833	44%	12%	10%	9%	13%	0.88 (0.83-0.91)	0.87 (0.84-0.92)
41-50, male	46,325	42%	9%	13%	9%	11%	0.81 (0.77-0.84)	0.80 (0.78-0.85)
51-60, male	130,236	41%	9%	13%	7%	12%	0.79 (0.74-0.80)	0.77 (0.76-0.82)
> 60, male	239,348	35%	7%	13%	6%	9%	0.62 (0.59-0.64)	0.61 (0.59-0.64)
Workload^c^ in quartiles								
< 5675	475,569	44%	12%	13%	9%	11%	1.00 (reference)	1.00 (reference)
5675-6354	514,270	45%	11%	14%	8%	12%	1.02 (1.02-1.04)	1.03 (1.01-1.02)
6355-7189	535,857	44%	10%	14%	8%	12%	1.01 (0.99-1.00)	1.00 (1.00-1.02)
> 7190	543,877	43%	9%	15%	7%	12%	0.99 (0.95-0.96)	0.96 (0.98-1.00)

^a^Adjusted for patient age and sex, geographical region, city size, GP age, sex and workload (except when stratified by this variable)

^b^Excluding 1,051 (52%) GPs working in partnership-practices due to lack of data on age and sex for this practice type

^c^Defined as annual number of ordinary consultations per 1,000 registered patients

Use of POCT varied across health care regions. Compared with patients living in the Capital Region of Denmark, patients in the North Denmark Region were more likely to receive POCT (adjusted OR = 1.19, 95% CI 1.18-1.20), particularly due to a higher use of urine test strips (16% vs. 12%) and CPR measurements (15% vs. 10%). Patients in the Central Denmark Region (adjusted OR = 0.81, 95% CI 0.80–0.82) and Region Zealand (adjusted OR = 0.91–0.92) were less likely to be tested compared with patients in the Capital Region. In contrast, there was no important rural–urban gradient, i.e., use of POCT varied little by the size of the city where the practice was located (Table 1).

Analyses restricted to GPs working in single-handed practices showed that compared with young female GPs (<41 years), female GPs aged 42–50 years were more likely to use POCT (adjusted OR = 1.06, 95% CI 1.02–1.11) while female GPs aged older than 60 years were less likely (adjusted OR = 0.84, 95% CI 0.80–0.86). Male GPs used less POCT compared with female GPs with the odds of POCT decreasing with increasing age (Table 1). The lowest use of POCT (except for use of urine test strips) was observed for male GPs older than 60 years versus female GPs < 41 years (adjusted OR = 0.61, 95% CI 0.59-0.64). In particular, a larger proportion of female GPs – except those older than 60 years – compared with male GPs used urine test strips prior to prescribing. Compared with GP age and sex, workload appeared to have little impact on odds of POCT and choice of specific tests (Table 1).

## Discussion

This nationwide population-based cross-sectional study covering a 10 year period and including more than 20 million antibiotic prescriptions issued by more than 2,000 Danish practices demonstrated an increasing use of POCT before antibiotic prescribing in Denmark from 2004–2013. In 2013, GPs carried out POCT before 44% of all antibiotic prescriptions. The pattern of POCT shifted over time towards relatively higher rates of CRP measurement in 2013. Nonetheless, there was wide variability in testing rates across GPs with a more than 5 fold inter-practice variation in the overall use of POCT and considerably more for the individual tests. Odds of POCT before antibiotic prescribing varied by patient age and sex, and young female GPs were more likely to test than older male GPs, whereas workload appeared to have little influence.

Our study has strengths and weaknesses. We included the entire Danish population through the use of nationwide registries and the study was conducted in a setting in which the National Health Service provides unfettered access to health care and partial reimbursement for prescribed medications, thus largely eliminating referral and diagnostic biases. The DNHSR holds detailed data on the practices and the GPs and track changes during the study period. The registry is used for reimbursement for the GPs from the national health insurance and is considered to provide almost complete information. We do not have estimates of the validity of the activities, but it is generally considered to be high [29]. Notwithstanding, we were unable to distinguish GP consultations during ordinary working hours and out-of-hours GP service, which is a main limitation of our study since these entities may differ substantially in the propensity for POCT. Moreover, we lacked data on GP age and sex working in partnership-practices. In Denmark, antibiotics are available on prescription only and a very small proportion is dispensed in hospitals. Since 2004, nationwide prescription data for antibiotic drugs have been assessable through the DHSPD. However, this registry only covers reimbursed medications and we likely underestimated the volume of tetracycline and quinolones since they were not reimbursed in 2013. According to national statistics the volume of tetracycline prescribed in the primary health care sector in 2013 amounted to 2.0 DDD per 1,000 inhabitants per day while the volume of quinolones was only 0.5 DDD per 1,000 inhabitants per day [32]. Another limitation of our study is that we do not know the indications for POCT nor the result. We used a 14-day time window to assess the use of POCT prior to antibiotic prescribing, and, in theory, the test could be unrelated to the actual antibiotic prescription. Nonetheless, we consider these events to be part of the same infectious episode. Moreover, as we only examined use of POCT in patients prescribed antibiotics, we cannot estimate to which extent use of POCT lead to non-prescribing decisions. Finally, we did not have data on practice staffing which may also influence POCT usage.

Limiting both over and under use of antibiotics in primary care is vital in reducing antibiotic resistance without exposing patients to unnecessary risks. Some of the high use of antibiotics in primary care may relate to diagnostic uncertainty [6]. GPs may be under pressure from patients who believe they need antibiotics [33]. With a negative test result at hand it may be easier for the GP to refuse prescribing an unnecessary antibiotic [15, 34, 35]. Thus, use of POCT to rule out a possible bacterial infection may reduce diagnostic uncertainty and increase confidence and acceptance of non-prescription decisions [36]. In a Dutch RCT Cals et al. [18] found that GPs assigned to CRP testing prescribed fewer antibiotics compared with those who did not use the test (31% vs. 53%). In line with this, other studies have shown that use of POCT is associated with reduced antibiotic prescribing [18, 22, 37–39] Importantly, a recent Cochrane review concluded that this reduction in antibiotic use in patients exposed to POCT is not associated with differences in recovery or duration of illness [38].

In Denmark, GPs have in-house lab facilities and potentially have a financial incentive to carry out POCT, because they are reimbursed by the National Health Service for the use of these tests. Qualitative studies have shown that GPs and patients generally report good acceptability of POCT [35, 40, 41] and economic analyses show that POCT is cost-effective [42, 43]. Increased use of CRP measurements and bacteriological cultures were the main drivers of the increased POCT, whereas urine test remained stable and GAS antigen swabs decreased. Compared with CRP measurements, bacteriological cultures requires overnight incubation and therefore cannot support “real-time” decisions. In this respect, the increase in bacteriological cultures may appear as a surprise. On the other hand, the increase could reflect cultures taken following a positive CRP test in order achieve a diagnosis and antimicrobial susceptibility. We also observed wide variability in the use of POCT across Denmark’s health care regions and in particular by GP age and sex. Potential explanations for the observed differences include a preference for female GPs by young female patients and families with children. It is also likely that older GPs feel more confident with their clinical judgement, than younger GPs. Qualitative research suggest that some GPs have concerns about over reliance on test results, e.g., false positive results that may lead to unnecessary antibiotic prescriptions as well as false negatives leading to lack of necessary antibiotic treatment [35]. Interviews with 66 GPs who participated in a RCT conducted in 6 European countries revealed that the GPs felt that CRP testing was most helpful in situations of uncertainty [44]. Thus, while helpful, the GPs indicated that they would restrict the use to cases of diagnostic uncertainty because of the time taken to obtain a result [44]. They also expressed concern that POCT could lead to more consultations in future [44, 45]. In a Swedish study, 49% of all Strep-A tests were performed in patients with a diagnosis of common cold, sinusitis, acute bronchitis or pneumonia, indicating that the tests were performed in patients with a very low probability of GAS [46]. Likewise, another Swedish study reported that 42% of all patients with a diagnosis of respiratory infection were examined with a CRP test [47]. The majority of these tests (69%) were performed in patients with a diagnosis of upper respiratory infection. These findings indicate that the tests were used too liberal and often not in a population with a high probability of bacterial infection.

## Conclusions

In conclusion, Danish GPs use POCT increasingly prior to antibiotic prescribing. In particular, the use of CRP measurement and bacteriological cultures increased substantially while the use of strep-A tests decreased. The proportion of patients tested and choice of test varied greatly across GPs.
